# The Mitochondrial Protein VDAC1 at the Crossroads of Cancer Cell Metabolism: The Epigenetic Link

**DOI:** 10.3390/cancers12041031

**Published:** 2020-04-22

**Authors:** Zohar Amsalem, Tasleem Arif, Anna Shteinfer-Kuzmine, Vered Chalifa-Caspi, Varda Shoshan-Barmatz

**Affiliations:** 1 Department of Life Sciences, Ben-Gurion University of the Negev, Beer-Sheva 84105, Israel; zohar2508@gmail.com (Z.A.); tashu100@gmail.com (T.A.); shteinfe@post.bgu.ac.il (A.S.-K.); 2 National Institute for Biotechnology in the Negev, Ben-Gurion University of the Negev, Beer-Sheva 84105, Israel; veredcc@bgu.ac.il

**Keywords:** cancer, histones epigenetics, metabolism, mitochondria, VDAC1

## Abstract

Carcinogenesis is a complicated process that involves the deregulation of epigenetics, resulting in cellular transformational events, such as proliferation, differentiation, and metastasis. Most chromatin-modifying enzymes utilize metabolites as co-factors or substrates and thus are directly dependent on such metabolites as acetyl-coenzyme A, S-adenosylmethionine, and NAD+. Here, we show that using specific siRNA to deplete a tumor of VDAC1 not only led to reprograming of the cancer cell metabolism but also altered several epigenetic-related enzymes and factors. VDAC1, in the outer mitochondrial membrane, controls metabolic cross-talk between the mitochondria and the rest of the cell, thus regulating the metabolic and energetic functions of mitochondria, and has been implicated in apoptotic-relevant events. We previously demonstrated that silencing VDAC1 expression in glioblastoma (GBM) U-87MG cell-derived tumors, resulted in reprogramed metabolism leading to inhibited tumor growth, angiogenesis, epithelial–mesenchymal transition and invasiveness, and elimination of cancer stem cells, while promoting the differentiation of residual tumor cells into neuronal-like cells. These VDAC1 depletion-mediated effects involved alterations in transcription factors regulating signaling pathways associated with cancer hallmarks. As the epigenome is sensitive to cellular metabolism, this study was designed to assess whether depleting VDAC1 affects the metabolism–epigenetics axis. Using DNA microarrays, q-PCR, and specific antibodies, we analyzed the effects of si-VDAC1 treatment of U-87MG-derived tumors on histone modifications and epigenetic-related enzyme expression levels, as well as the methylation and acetylation state, to uncover any alterations in epigenetic properties. Our results demonstrate that metabolic rewiring of GBM via VDAC1 depletion affects epigenetic modifications, and strongly support the presence of an interplay between metabolism and epigenetics.

## 1. Introduction

Cancer cells within a tumor adapt their metabolism to fulfill their immediate bioenergetic and biosynthetic needs for cell growth, proliferation, and the production of effector molecules [1,2]. This adaptation of cancer cells to their oncogenic program and the requirements of cancer growth leading to metabolic addiction, is influenced by their microenvironment. 

As for normal cells, the transcriptional network of cancer cells establishes a specific molecular program that is in-tune with nutritional conditions [3,4,5]. It has been well demonstrated that cellular metabolic activity regulates transcriptional networks and cell-fate decisions via epigenetic programs involving metabolite-dependent effects on chromatin organization. Epigenetics refers to a change in chromatin that leads to the regulation of gene expression without alterations in the DNA sequence [6]. Epigenetic modifications include DNA methylation, and/or histone modifications by acetylation, ubiquitination, methylation, phosphorylation, sumoylation, glycosylation, and biotinylation, changes that play a critical role in many cellular processes [7,8,9,10,11,12,13]. The enzymes responsible for these modifications include histone acetyltransferases (HATs), histone deacetylases (HDACs), methyltransferases (KMTs), and demethylases (KDMs) [14,15,16].

There is now an accumulation of evidence that the epigenome is sensitive to cellular metabolism and a link between metabolism and epigenetics [3,4,5,17,18,19] has been proposed, with epigenetics and gene transcription being influenced by products of metabolic pathways [20]. Indeed, many of the chemical modifications that decorate DNA and histones are adducts derived from the intermediates of cellular metabolic pathways, and a number of the enzymes that can remove these marks use metabolites in the reactions they catalyze. Moreover, chromatin-modifying enzymes are directly dependent on metabolites such as acetyl-CoA, S-adenosylmethionine (SAM) and NAD+. Therefore, these nuclear activities are directly influenced by the nutritional status of the cell.

Histone acetylation is emerging as a major regulatory mechanism thought to modulate gene expression by altering the accessibility of transcription factors to DNA [21,22]. The main sites of acetylation include at least four highly-conserved lysines in histone H4 (K5, K8, K12, and K16) and five in histone H3 (K9, K14, K18, K23, and K27), as well as less-conserved sites in histones H2A and H2B. Histone acetylation is sensitive to overall acetyl CoA levels. In mammalian cells, three major enzymes generate acetyl CoA, namely acetate-dependent acetyl-CoA synthetase 2 (ACSS2), citrate-dependent ATP-citrate lyase (ACLY), and the mitochondrial pryruvate dehydrogenase complex (PDC). Upon metabolic insult, functional PDC translocates to the nucleus, where it generates a nuclear pool of acetyl CoA that increases histone acetylation [23]. 

Histone modifications by methylation, are also a fundamental feature of human malignancies [24]. Histone methylation can occur on lysine or arginine residues, with these histone methyl marks either activating or repressing gene expression [25]. Lysine methylation of H3 and H4 is implicated in both transcriptional activation and repression, depending on the methylation site, while arginine methylation promotes transcriptional activation [26]. Methylation is linked to the intermediary metabolism through SAM, the primary source of methyl groups generated in the folate and methionine cycles, coupled to serine-derived one-carbon metabolism [27,28,29]. The activities of both histone methyltransferases (HMT) and DNA methyltransferases (DNMT) depend on intracellular SAM levels, which vary according to the nutrient availability of serine and methionine. 

Interplay between metabolism and epigenetics [4,5,7,8,9,12,13,17,18,19,30] is now considered an enabling characteristic of cancer [4,31,32]. Analysis of cancer samples revealed genome-wide epigenetic alterations that potentially regulate gene expression and are associated with tumor progression [33]. For example, in glioblastoma (GBM), the metabolic enzymes isocitrate dehydrogenase 1 (IDH1) and pyruvate kinase M2 (PKM2) were proposed as the link between tumor metabolism and epigenetics. Specifically, both IDH1 and PKM2 directly influence DNA hyper-methylation and histone modification. For example, EGFR-mediated phosphorylation of PKM2 initiates PKM2 translocation to the nucleus, allowing it to interact and phosphorylate histone 3 at a threonine residue [34]. A decline in glucose metabolism that accompanies differentiation is associated with decreased acetyl-CoA availability and reduced histone acetylation [35]. In this context, mitochondrial metabolism has emerged as a key target for cancer therapy [36,37].

The mitochondrial protein VDAC1, is a key regulator of metabolic and energy homeostasis that contributes to the metabolic phenotype of cancer cells [38]. The location in the outer mitochondrial membrane (OMM) allows it to serve as the channel that mediates the flux of ions, nucleotides, and other metabolites up to ~5000 Da (e.g., pyruvate, malate, succinate, and nucleotides NADH/NAD) across this membrane [39,40,41]. VDAC1 facilitates shuttling of ATP/ADP and NAD+/NADH, with mitochondria-generated ATP being transported to the cytosol in exchange for ADP, which is utilized in oxidative phosphorylation (OXPHOS) to generate ATP. The protein is also involved in cholesterol transport and mediates the fluxes of ions, including cytosolic and Ca^2+^, as well as being involved in mitochondria-ER Ca^2+^ signaling, serving as a ROS transporter, and regulating the redox states of mitochondria and the cytosol [40,42]. Furthermore, recently we demonstrated that VDAC1 oligomers mediate the release of mitochondrial DNA fragments [43]. Thus, VDAC1 appears to be a convergence point for a variety of cell survival and death signals, mediated through association with various ligands and proteins [40,42]. 

The importance of VDAC1 in cell energy and metabolism homeostasis is reflected in its over-expression in many cancers [44,45], and with down-regulation resulting in reduced metabolite exchange between mitochondria and cytosol and inhibited cell and tumor growth [44,46,47,48]. We previously reported that siRNA specific for human VDAC1 (si-VDAC1) inhibited solid tumor development and growth in cervical and lung cancers and triple negative breast cancer [44,46,47], as well as in GBM [49]. Recently, we also demonstrated that the residual tumor left after si-VDAC1 inhibition of tumor growth, exhibited a reversal of the oncogenic properties, such as reprogramed metabolism, angiogenesis, epithelial–mesenchymal transition (EMT), invasiveness, and stemness, leading to differentiation into neuron- and astrocyte-like cells [49]. Depleting VDAC1 caused alterations in the transcription factors (TFs) that regulate signaling pathways associated with cancer hallmarks, and modified the expression of over 4000 genes [49]. 

In this study, we investigated whether the effects of VDAC1 depletion on tumor cells are mediated via a metabolism–epigenetics axis. To this end, we used DNA microarrays, q-PCR, and specific antibodies to analyze the effects of si-VDAC1 treatment of glioblastoma tumors on the expression of epigenetic-related genes. Following depletion of VDAC1 in cancer cells in a glioblastoma tumor, we detected epigenetic alterations in the level of histone modifications [50,51,52] at the methylation and acetylation states and in epigenetic-related enzyme expression levels. These findings support the interactions between metabolism and changes in epigenetics.

## 2. Materials and Methods

### 2.1. Materials

The cell transfection agents JetPRIME and JetPEI were obtained from PolyPlus transfection (Illkirch, France), while non-modified and 2′-O-methyl-modified hVDAC1-siRNAs were obtained from Genepharma (Suzhou, China). Triton X-100, hematoxylin, and eosin were obtained from Sigma (St. Louis, MO, USA). Paraformaldehyde was purchased from Emsdiasum (Hatfield, PA, USA). Primary antibodies, their source, and the dilutions used are detailed in Table S1. Horseradish peroxidase (HRP)-conjugated anti-mouse, anti-rabbit, and anti-goat antibodies were from KPL (Gaithersburg, MD, USA). 3,3-diaminobenzidine (DAB) was obtained from (ImmPact-DAB, Burlingame, CA, USA).

### 2.2. Xenograft Experiments 

U-87MG glioblastoma cells (2 × 10^6^) were inoculated s.c. into the hind leg flanks of athymic eight-week-old male nude mice (Envigo, Israel). Eleven days post-inoculation, tumor volume was measured (50–80 mm^3^) and mice were randomized into two groups (9 animals/group), treated with non-targeting siRNA (si-NT) or si- hVDAC1 (S: 238-5′-ACACUAGGCACCGAGAUUA-3′-256 and AS: 238-5′-UAAUCUCGGUGCCUAGUGU-3′) mixed with in vivo JetPEI reagent (50 nM final concentration, 2 boluses) every three days. At the end of the experiments, the mice were sacrificed, tumors were excised, and half of each tumor was either fixed and processed for IHC or frozen in liquid nitrogen for later immunoblot and RNA isolation. Experimental protocols were approved by the Institutional Animal Care and Use Committee (IL-01-08-2014). 

### 2.3. Gel Electrophoresis and Immunoblotting 

Tumor tissues were solubilized to extract proteins for gel electrophoresis and immunoblotting that were carried out as described in the Supplementary Data Section. 

### 2.4. Immunohistochemistry (IHC) 

Immunohistochemical staining was performed on formalin-fixed and paraffin-embedded tumors obtained from si-NT- and siRNA specific to human VDAC1 (si-hVDAC1)-treated tumors as described previously [49] and in the Supplementary Data Section. The antibodies used are listed in Table S1. 

### 2.5. RNA Preparation, DNA Microarray Analysis, and Quantitative Real-Time PCR (q-RT-PCR) 

Total RNA isolation from si-NT- and si-hVDAC1-treated tumors, Affymetrix whole transcript expression microarray analyses, and quantitative real-time PCR (q-RT-PCR) using specific primers (Table S1) were carried out as described in the Supplementary Data Section.

### 2.6. Liquid Chromatography High-Resolution Mass Spectrometry (LC-HR-MS/MS) Analysis

Proteins for LC-HR MS/MS were extracted from the tumor tissues using a lysis buffer (100 mM Tris-HCl, pH 8.0, 5 mM DTT 4% SDS and a protease inhibitor cocktail (Calbiochem, San Diego, CA USA), followed by homogenization, incubation for 3 min at 95 °C, and centrifugation (10 min, 15,000× *g*). Samples were stored at −80 °C until MS/MS analysis. The LC-HR MS/MS analysis was performed as a service in the Nancy and Stephen Grand Israel National Center for Personalized Medicine, Weizmann Institute, as described in the Supplementary Data Section. LC-HR-MS/MS data were imported into Partek Genomics Suite software (Partek, St. Louis, MO, USA) and differences between expression levels of the proteins in the different groups were calculated using a *t*-test. Functional enrichment analysis of differentially-expressed proteins was performed using the DAVID and Gene Ontology (GO) bioinformatics resources, v6.7 [53]. 

### 2.7. Statistics and Data Analysis 

The means ± SE of results obtained from three independent experiments are presented. *t*-test was employed to evaluate significant differences between the experimental groups. *P-*values were: p* < 0.05, *p* **< 0.01, and *p* *** < 0.001. Significance was also analyzed using a non-parametric Mann–Whitney *U* test to compare control and experimental groups, with Statistica 13.1 software.

## 3. Results 

In previous studies [44,46,47], we demonstrated that nano-molar concentrations of a single siRNA specific to human VDAC1 (si-hVDAC1), silenced VDAC1 expression both in vitro and in vivo, and inhibited the growth of various types of solid tumors. Recently [49], we demonstrated that si-hVDAC1 inhibits GBM tumor growth, and that the residual tumor cells exhibit a reversal of their oncogenic properties, with inhibition of the reprogramed metabolism, angiogenesis, EMT, invasiveness, and stemness. This reprograming involves alterations in TFs and expression of multiple genes that regulate signaling pathways associated with cancer hallmarks. Here, based on the proposed link between metabolism and epigenetics [3,4,5,17,18,19], we addressed the involvement of epigenetics in the interplay between reprograming metabolism and the changes in the oncogenic signaling networks observed upon VDAC1 depletion.

### 3.1. VDAC1 Depletion by si-RNA against Human (h)VDAC1 Inhibits Tumor Growth and Reprogramed Metabolism of U-87-MG Cell Line-Derived Tumors

**Figure 1:**
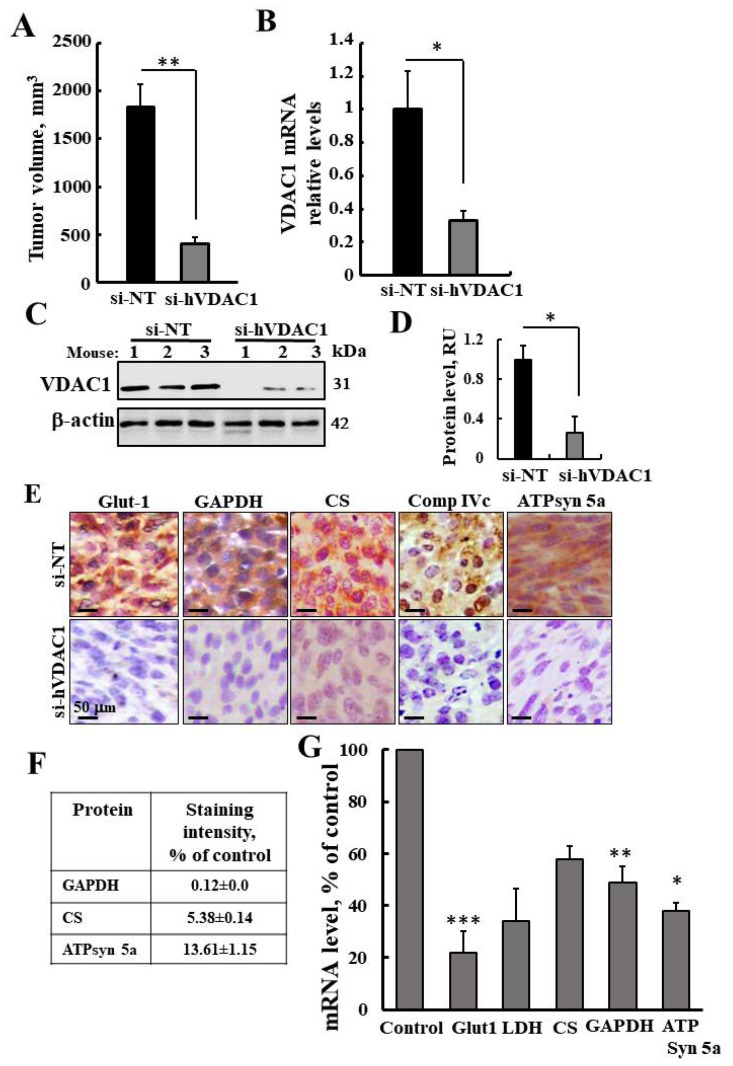
VDAC1 depletion by specific si-RNA inhibits tumor growth and reprogrammed metabolism of U-87-MG cell-derived tumors. (**A**) U-87-MG cells were inoculated subcutaneously into nude mice (3 × 10^6^ cells/mouse). When the tumor volume reached 60–100 mm^3^, the mice were divided into two groups (five mice per group) and treated with non-targeted siRNA (si-NT) or human VDAC1-specific si-RNA (si-hVDAC1) by intratumoral injection (every 3 days) to a final concentration of 75 nM per tumor. The calculated average tumor volume (means ± SEM, ** *p* < 0.01) are presented in mm^3^. (**B**,**C**) VDAC1 mRNA expression levels in si-NT-TTs and si-hVDAC1 were analyzed by qRT-PCR (B) or immunoblotting (C). (**D**,**E**) Expression of selected metabolism-related proteins (Glut1, GAPDH, citrate synthase (CS), complex IV, and ATP Syn5a), as analyzed by immunohistochemical (IHC) staining using specific antibodies (**F**) and qRT-PCR-assessed mRNA levels (**G**) of si-NT- or si-hVDAC1-TTs. *p* < 0.001 (***), *p* < 0.01 (**), *p* < 0.05 (*).

Subcutaneous (s.c.) U-87MG-derived xenografts were established in athymic nude mice, and when the tumor volume reached 50–100 mm^3^, the mice were split into two tumor-volume-matched groups and treated intratumorally with non-targeting si-RNA (si-NT) or with si-hVDAC1-2/A. A decrease of 77% in tumor volume was obtained (Figure 1A) with si-hVDAC1-2/A treatment. The level of VDAC1 in the si-NT- and si-VDAC1-2/A-treated tumors (TTs) was analyzed by qRT-PCR (Figure 1B) and immunoblotting (Figure 1C,D and Figure S2A), showing a decrease of 70% and 75%, respectively. 

Next, the expression levels of metabolism-related enzymes including the glucose transporter (Glut-1), glyceraldehyde dehydrogenase (GAPDH), and lactate dehydrogenase (LDH), the Kreb’s cycle enzyme, citrate synthase (CS), the mitochondrial electron transport complex IVc, and ATP synthase 5a (ATPsyn5a) were analyzed in the s-NT-TTs and si-VDAC-TTs using IHC (Figure 1E,F) and qPCR (Figure 1G). The results clearly showed that the expression levels of all tested proteins were reduced in si-hVDAC1-TTs, consistent with alterations in glycolysis and oxidative phosphorylation (OXPHOS). 

### 3.2. VDAC1 Depletion by si-hVDAC1-Induced Alteration of the Gene Expression Profile of si-hVDAC1-TTs 

**Figure 2:**
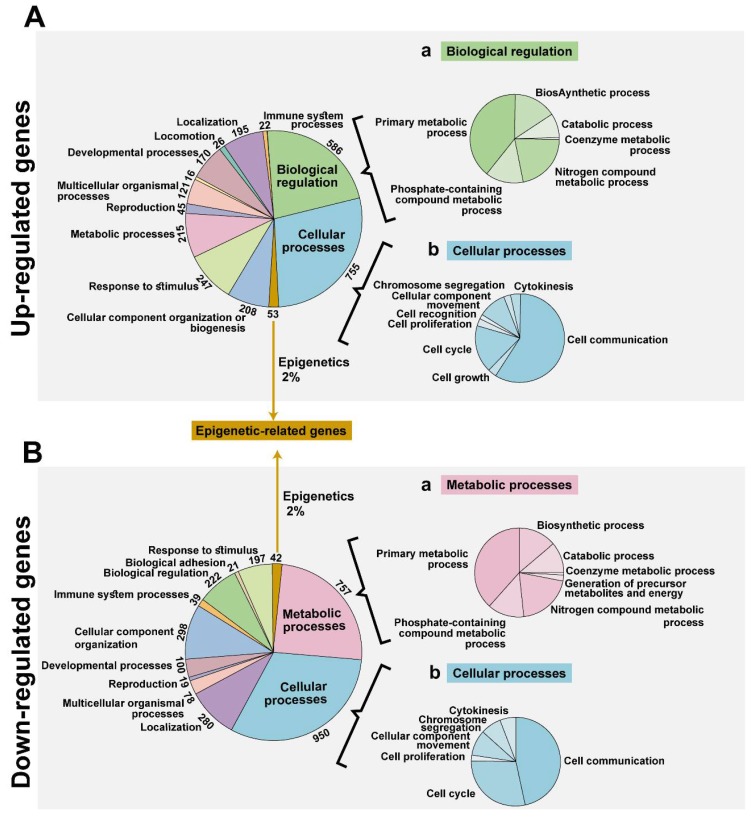
Functional analysis of genes differentially expressed in si-hVDAC1-TTs. RNA, isolated from tumors treated with si-hVDAC1 or si-NT (75 nM), was subjected to Affymetrix DNA microarray and bioinformatics analyses. This revealed alterations in about 5271 genes (fold change > 2, FDR-adjusted *p*-value < 0.05), of which 2980 genes were up-regulated (**A**) and 2291 were down-regulated (**B**). Functional analysis was based on the Gene Ontology (GO) system. Significantly-enriched pathway-associated genes differentially expressed in si-hVDAC1-TTs are listed, with the number of genes related to a given pathway indicated inside the chart. The major gene groups associated with biological regulation and cellular processes from the up-regulated are further presented as a sub-group (**A**(**a**,**b**)). The major gene groups that were down-regulated, cellular processes, and metabolic processes were further presented with their sub-group (**B**(**a**,**b**)). The epigenetic processes-related genes numbered 95 genes with the 53 genes up-regulated (2%) and 42 genes down-regulated (2%) are indicated.

Affymetrix DNA microarray analysis of the gene expression profile of si-hVDAC1-TTs and si-NT-TTs (Figure 2) revealed 5271 significantly-changed genes (≥2-fold change, false discovery rate < 0.05), with 2291 genes down-regulated and 2980 genes up-regulated in the si-hVDAC1-TTs. The differentially-expressed genes in the si-hVDAC1-TTs-treated tumors are also presented as a volcano plot (Figure S1) Functional analysis based on the Gene Ontology (GO) system revealed alterations in key functions and pathways including metabolic, biosynthetic, and developmental processes, biological regulation, and epigenetic processes among many others as presented in Figure 2. The major functional groups were the cellular processes-related genes, with 755 genes up-regulated (29%, Figure 2(Ab)) and 950 (32%, Figure 2(Bb)) down-regulated in the si-hVDAC1-TTs. Metabolism-related genes were also affected with 215 (8.3%, Figure 2A) up-regulated and 757 (26%, Figure 2(Ba)) down-regulated genes in the si-hVDAC1-TTs, as presented with their subgroups. Among the biological regulation-related genes, 586 (23%, Figure 2(Aa)) genes were up-regulated and 222 (7.6%) were down-regulated in the si-hVDAC1-TTs. Another interesting group of genes that is the focus of this study, the epigenetic-related genes, were differentially expressed between si-hVDAC1-TTs and si-NT-TTs, with 2% being up-regulated and 2% down-regulated (Figure 2). 

**Figure 3:**
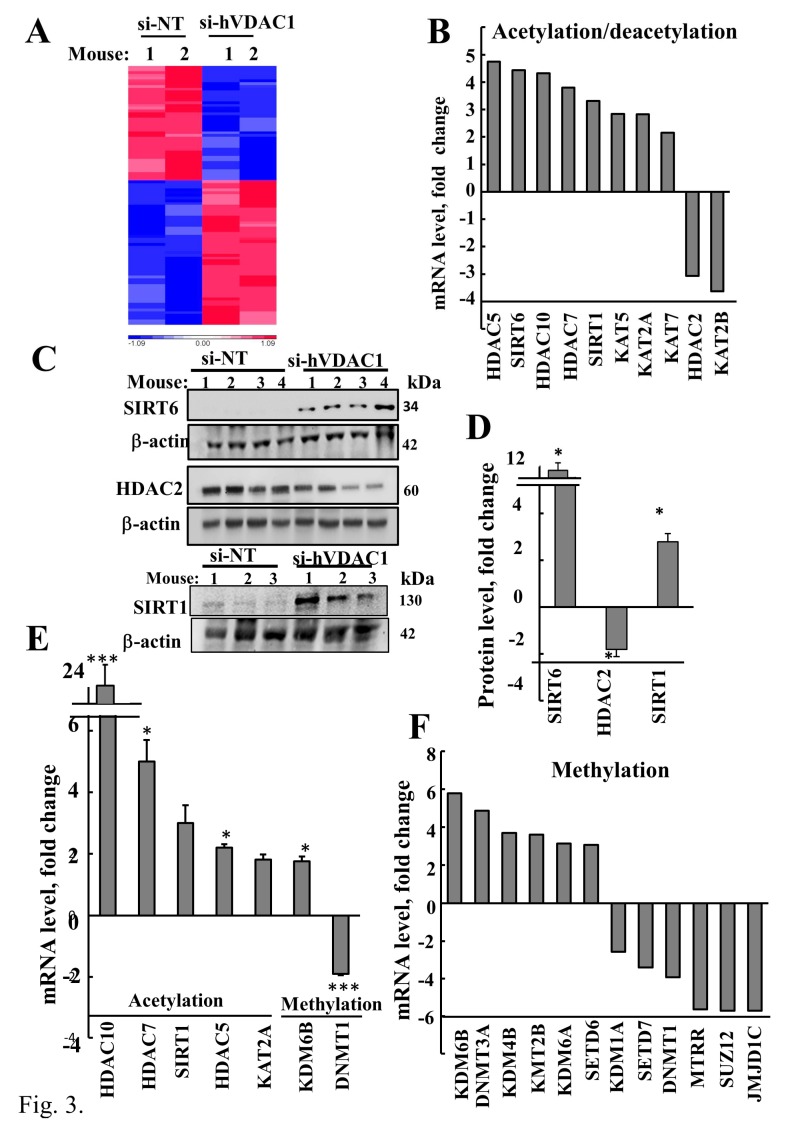
VDAC1 depletion altered the expression of epigenetics-related genes and proteins. (**A**) Hierarchical clustering of selected epigenetics-related genes (95) which were significantly up- or down-regulated in si-hVDAC1-TTs, relative to si-NT-TTs (FDR-adjusted *p*-value < 0.05 and linear fold change of decreased or increased expression > 2). The color scale of the standardized expression values is shown. (**B**) Fold change of genes associated with histone acetylation/deacetylation (selected from DNA microarray data, Table 1 and Table 2). Immunoblotting of the acetylation-related enzymes SIRT6, HDAC2, and SIRT1 using specific antibodies (**C**) and quantification of the blots (**D**). (**E**) DNA microarray analysis of up- or down-regulated methylation-related genes in si-hVDAC1-TTs, relative to si-NT. (**F**) mRNA levels of selected proteins from (**B**,**E**) were validated by qRT-PCR using specific primers. Results show means ± SEM (*n* = 3). *p* < 0.001 (***), *p* < 0.05 (*).

A hierarchical clustering of the up- and down-regulated epigenetic-related genes is presented in Figure 3A and their fold change, *p*-value, and function are described in Table 1 and Table 2. 

The expression levels of selected genes were further investigated in si-hVDAC1-TTs and si-NT-TTs as presented below.

### 3.3. VDAC1 Depletion Altered Acetylation and Methylation of Histone 3 and Histone 4 

Histone modifications at various lysine residues are one of the epigenetic signatures, where the acetylated, none-acetylated, methylated, or non-methylated state marks the gene for activation or suppression, thereby enabling the dynamic and reversible regulation of transcription [82]. The sites of acetylation include at least four highly-conserved lysines in histone H4 (K5, K8, K12, and K16), and five in histone H3 (K9, K14, K18, K23, and K27), as well as less-conserved sites in histones H2A and H2B.

We analyzed the expression levels of the histone deacetylases (HDACs) including HDAC2, HDAC5, HDAC7, and HDAC10 in si-hVDAC1-TTs and si-NT-TTs. With the exception of HDAC2, all were increased (3- to 5-fold) in the si-VDAC1-TTs, as revealed by the DNA microarray data (Figure 3B, Table 1), immunoblotting (Figure 3C,D and Figure S2D), and q-RT-PCR (Figure 3F).

Similarly, the levels of the class III histone deacetylases, sirtuins [83], SIRT1 (Figure S2C), and SIRT6 (Figure S2B), the NAD+-dependent lysine deacetylase that deacetylates histones and non-histone proteins, were also increased in si-hVDAC1-TTs (Figure 3B–E, Table 1). 

Interestingly, DNA microarray data indicated differential regulation of the lysine (K)-acetyl-transferases (KATs) where the expression levels of KAT5, KAT2A (also known as GCN5**),** and KAT7 were increased, while that of KAT2B was decreased in the si-hVDAC1-TTs (Figure 3B, Table 1).

A similar dichotomy was found in the expression levels of enzymes associated with methylation and de-methylation. The expression levels of the lysine demethylases KDM6B, KDM4B, and KMT2B, and the DNA methyltransferase, DNMT3A and SET domain containing 6 (SETD6), were increased in the si-hVDAC1-TTs (Figure 3C, Table 2). In contrast, the levels of lysine demethylase 1A (KDM1A), DNA methyltransferase 1 (DNMT1), methionine synthase reductase (MTRR), polycomb repressive complex 2 subunit (SUZ12), jumonji-domain-containing 1C (JMJD1C), and SET-domain-containing lysine methyltransferase 7 (SETD7) were decreased (Figure 3E,F, Table 2).

**Figure 4:**
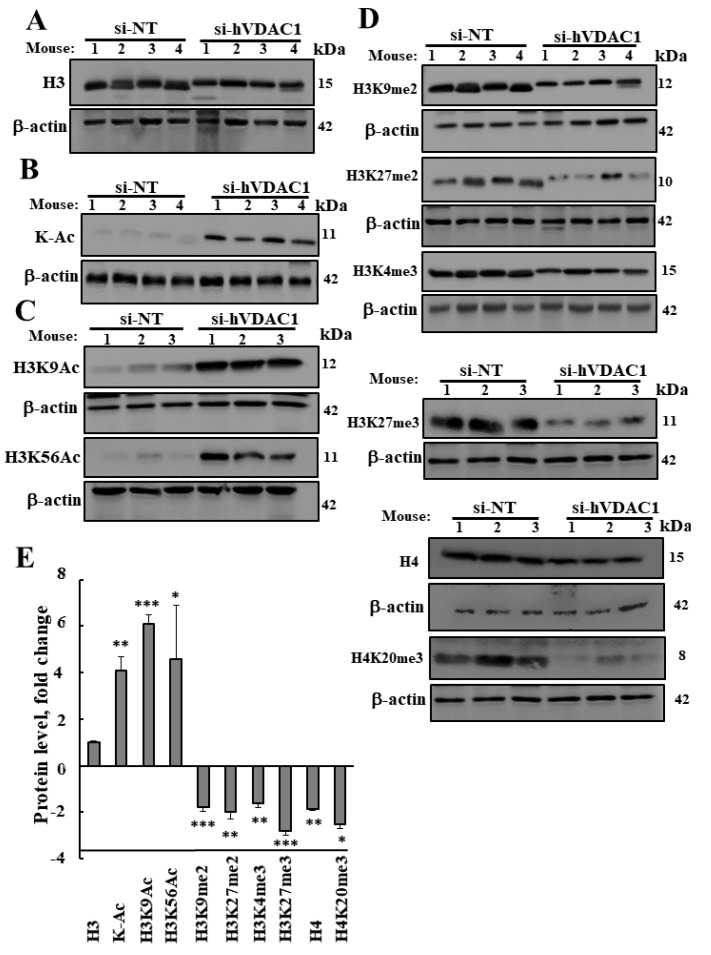
VDAC1 depletion reprograms epigenetics-related modifications. Proteins were isolated from si-NT-TTs and si-hVDAC1-TTs in the presence of deacetylation inhibitors as described in Materials and Methods. Immunoblots of histone 3 (H3) (**A**), and of acetylated histones (K-Ac) (**B**) and of H3K9 and H3K56 (**C**) using specific antibodies are shown. β-actin served as an internal loading control. (**D**) Di-methylation at H3K9 (H3K9me2) and H3K27 (H3K27me2) and tri-methylation at H4K3 (H3K4me3), H3K27 (H3K27me3), and H4K20 (H4K20me3). (**E**) Quantification of the blots shown in (**A**–**D**). Results show means ± SEM (*n* = 3). *p* < 0.001 (***), *p* < 0.01 (**), *p* < 0.05 (*).

Next, we focused on the effects of VDAC1 silencing on specific histone modification by acetylation or methylation at a specific lysine (Figure 4). To avoid deacetylation, tumor proteins were extracted in the presence of deacetylase inhibitors (sodium butyrate and sirtinol). Histone H3 levels, as revealed by immunoblot, were the same in si-NT-TTs and si-VDAC1-TTs (Figure 4A,E and Figure S3A). However, the electrophoretic mobility of H3 in the si-hVDAC1-TTs was slower and the bands appeared sharper (Figure 4A and Figure S3A). Since this could indicate different post-translational modifications in the two tumors the acetylation and methylation states were further investigated.

Results from antibodies directed against general acetylated lysine residues (K-Ac) indicated a dramatic increase in the acetylation level of the 12 kDa protein representing histones (Figure 4B,E and Figure S3B). Interestingly, although general acetylated lysine antibodies were used, the histone protein band was the major one and only a few additional bands with weak immune-reactivity were found (Figure S3B). 

With specific antibodies for histone H3 acetylation in specific lysine residues such as found in K9 (H3K9Ac) and K56 (H3K56Ac) the results indicated an essential absence of such acetylated proteins in the si-NT-TTs but a strongly increased signal (4- to 6-fold) in the si-hVDAC1-TTs (Figure 4C,E and Figure S3C,J).

Interestingly, in contrast to the acetylation, the methylation of histones 3 and 4 was reduced in the si-hVDAC1-TTs (Figure 4D,E and Figure S3D–F). This was demonstrated using antibodies specific for histone H3 methylation on specific lysine resides such as K4 with one-methyl (H3K4me), K9, with two-methyl groups, (H3K9me2), K27 with two-methyl groups (H3K27me2), or tri-methyl (H3K27me3) and of histone H4 methylated at K20 with tri-methyl (H4K20me3) (Figure 4D,E and Figure S3D–F). The level of H4 in the si-hVDAC1-TTs was decreased by 46% relative to the level in the si-NT-TTs (Figure 4D and Figure S3H).

**Figure 5:**
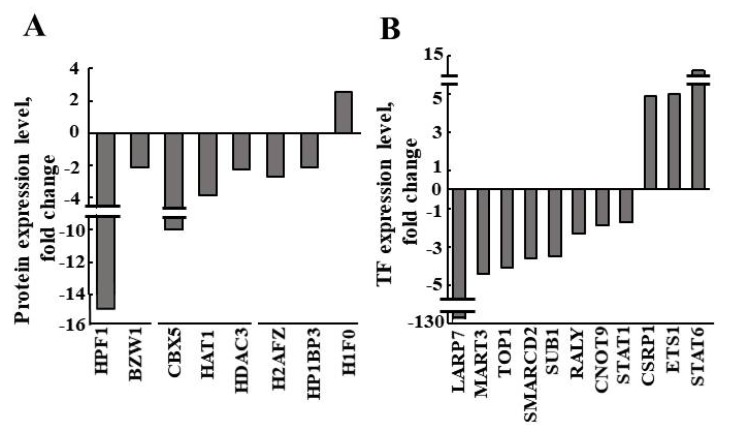
Epigenetics-related proteins differentially expressed in si-NT-TTs and si-hVDAC1-TTs revealed by LC-HR MS/MS analysis. (**A**,**B**) Quantitative analysis of proteins differentially expressed in si-VDAC1-TTs, in comparison to si-NT-TTs, using LC-HR MS/MS data and presented as fold change protein expression level. Up- or down-regulated histone-associated proteins (**A**) and transcription factors (**B**), with *p*-values presented in Table 3 and Table 4, respectively.

Finally, proteomics results, obtained by LC-HR MS/MS analysis of si-NT-TTs and si-hVDAC1-TTs, demonstrated that with the exception of one protein, the expression of all other epigenetic-related proteins was decreased in the si-hVDAC1-TTs relative to the level in the si-NT-TTs (Table 3, Figure 5A). These proteins included the histone PARylation factor 1 (HPF1) that promotes histone serine ADP-ribosylation, chromobox protein homolog 5 (CBX5) that binds to H3K9me, and histone acetyltransferase type B catalytic subunit (HAT1) that acetylates soluble histone H4 at K5 and K12, which were reduced, 14.9-, 9.2-, and 3.8-fold, respectively, in the si-hVDAC1-TTs (Table 3, Figure 5A). Interestingly, the expression level of the H1 histone family, member 0 (H1F0) in si-hVDAC1-TTs was increased. H1F10 is found in cells at terminal stages of differentiation or with low rates of cell division and the increase is in agreement with our suggestion that VDAC1 depletion leads to differentiation [49].

The proteomic data also revealed altered expression of a number of transcription factors (TFs) in the si-hVDAC1-TTs (Table 4, Figure 5B). These TFs included La-related protein 7 (LARP7), a negative transcriptional regulator of polymerase II genes that was decreased about 127-fold, and matrin-3 (MATR3), a protein thought to interact with other nuclear matrix proteins to form the internal fibrogranular network and thus play a role in transcription, which was decreased about 4.4-fold. The expression of other TFs such as cysteine- and glycine-rich protein 1 (CSRP1), which is involved in regulatory processes important for development and differentiation, ETS proto-oncogene 1 (ETS1), which controls the expression of cytokine and chemokine genes, the differentiation, survival and proliferation of lymphoid cells, and the signal transducer and activator of transcription 6 (STAT6), which serves a dual function of signal transduction and activation of transcription, were increased, by 4.9-, 5-, and 11.5-fold, respectively, in si-hVDAC1-TTs (Table 4, Figure 5B). 

Taken together, these results indicate that the VDAC1 depletion-mediated effects on GBM tumors involve alterations in the expression of about 5000 genes including those associated with re-programed metabolism and modifying the epigenetic landscape.

## 4. Discussion

Major cell decisions that require metabolic alterations and differential gene expression are often epigenetically-driven, with the metabolism–epigenetics link having been extensively explored in tumorigenesis [25,28]. Here, we compared the epigenetic profile of a GBM tumor and of a “residual tumor” obtained after si-hVDAC1 treatment, and which displayed a reversal of the oncogenic metabolism and other cancer-related properties, in order to identify alterations in the epigenetic landscape caused by cell depletion of VDAC1. 

**Figure 6:**
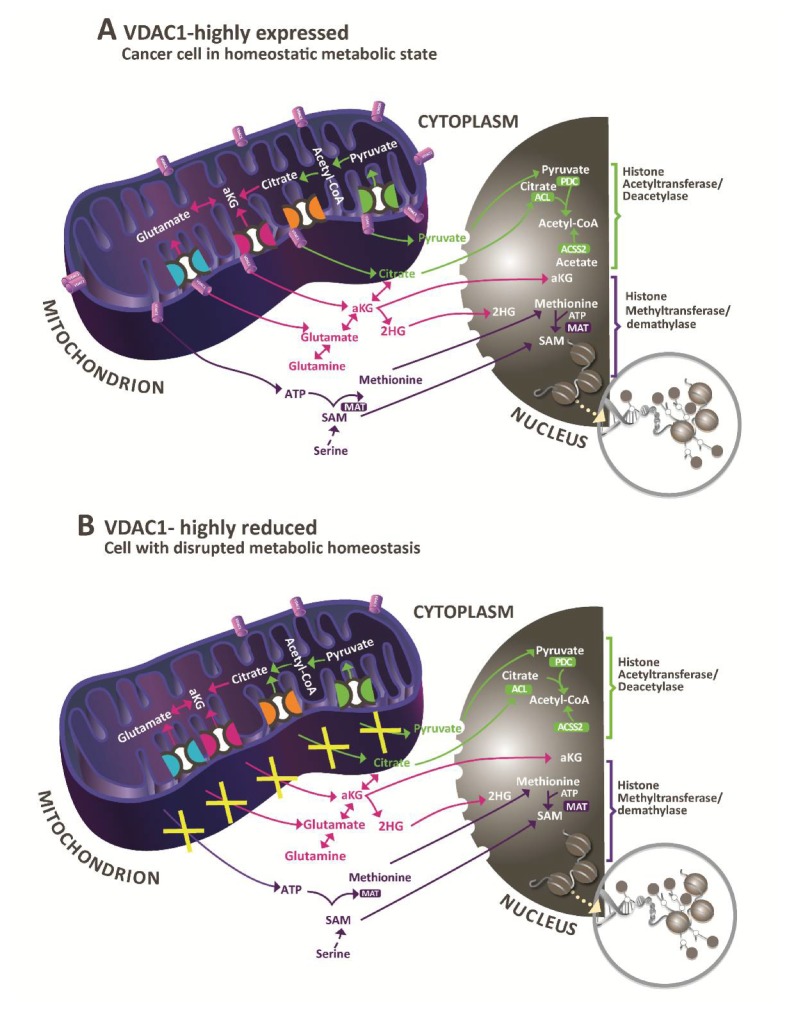
A simplified schematic depicting the interplay between mitochondrial VDAC1 metabolic pathways that provide substrates for enzymes that modify chromatin. (**A**) In cancer cells where the mitochondria have high levels of VDAC1, mitochondrial metabolic pathways generate carbon groups required for methylation (turquoise), acetylation (orange), or demethylation (dark blue) of chromatin. TCA cycle metabolites provide carbon units for both acetylation and demethylation via α-ketoglutarate (αKG). Metabolite transport across the inner mitochondrial membrane requires transporters that are shown. These metabolites, however, cross the outer mitochondrial membrane (OMM) via VDAC1 and the metabolites directly relevant to chromatin regulation reach the nucleus in which several metabolic enzymes are localized, including methionine adenosyl-transferase (MAT), ATP-citrate lyase (ACL), pyruvate dehydrogenase complex (PDC), and acetyl-CoA synthetase 2 (ACSS2). (**B**) Following VDAC1 depletion, there is a strong reduction in mitochondrial metabolism [44,46,47,48] due to the decrease in substrates transported into the mitochondria and the inability of the produced metabolites to exit the mitochondria. This limits the substrates for chromatin modifications. The scheme has been modified from [87].

VDAC1, by controlling mitochondrial activity, controls the accessibility of intermediate metabolites necessary for generating and modifying epigenetic marks in the nucleus. As summarized in Figure 6, these essential mediators of epigenetic processes such as NADH, citrate, pyruvate, ATP, acetyl CoA, and α-ketoglutarate need VDAC1 in order to exit from the mitochondria and reach the nucleus, thus controlling the metabolite pools for the epigenetic landscape. Here, we show that by altering mitochondrial function via VDAC1 silencing, we control such metabolite-induced epigenetic changes as histone acetylation and histone methylation, affecting the expression of about 2000 genes and having profound effects on cancer development. It has been shown that alterations in the metabolism affect molecular rewiring of cancer cells, facilitating cancer development and progression with the interplay between metabolomics and epigenetics, promoting neoplastic transformation [84]. 

Our findings, show for the first time, that VDAC1 via metabolic reprograming reversing the well-known metabolic reprograming of cancer cells [1,2], involves epigenetic remodeling.

### 4.1. Epigenetics, Gene Transcription, Metabolism, and VDAC1 

Epigenetics and cancer has been tightly linked where reprograming the transcriptional circuitry by remodeling the three-dimensional structure of the genome is exploited by cancer cells to promote tumorigenesis [85,86]. Epigenetic mechanisms underlie the phenotypic plasticity of cells, and provide the foundation for oncogenic transformation [25]. In addition, the metabolic state and chromatin structure are tightly linked, enabling gene expression to adapt to the changing environment and this differential gene expression is often epigenetically-driven [87]. 

Indeed, a growing body of evidence now suggests that the metabolism–epigenetics axis optimizes adaptive responses to changes in environment in normal (e.g., development and stem cell differentiation) and disease states (e.g., cancer), thus linking metabolic stress to cellular functions [4]. In brain tumors, metabolic and micro-environmental factors were shown to give rise to a convergence of epigenetic deregulation, with the aberrant epigenetic pathways subsequently affecting cell identity, cell state, and neoplastic transformation [88]. 

Bioenergetic pathways and enzymes provide metabolic co-factors, such as acetyl-CoA and S- adenosylmethionine (SAM), which serve as donor substrates for acetylation and methylation reactions, respectively. Both these processes are central to the regulation of chromatin (Figure 6). Global levels of nuclear histone acetylation are sensitive to overall acetyl CoA levels, produced in the mitochondria by fatty acid oxidation, and pyruvate oxidation mediated by pyruvate decarboxylase. Demethylation is also susceptible to metabolic fluctuations as it is regulated by histone and DNA demethylases whose activities are modulated by alpha-ketoglutarate (α-KG), fumarate, and succinate [89], three intermediate metabolites of the tricarboxylic acid (TCA) cycle. The production of these substrates and their translocation from the mitochondria to the nucleus requires VDAC1 and does not occur in the absence of the VDAC1 protein (Figure 6).

As a transporter of metabolites in the mitochondria, VDAC1 is a key regulator of the metabolic and energy homeostasis, contributing to the metabolic phenotype of cancer cells [38]. We have previously demonstrated that tumors depleted of VDAC1 undergo metabolic reprograming (Figure 1), leading to a reduction of tumor growth, invasiveness, and angiogenesis, and the disappearance of cancer stem cells, while inducing tumor cell differentiation [49]. Our results identified, an estimated 5000 genes whose expression was altered upon VDAC1 depletion [49] (Figure S1). These included genes associated with metabolism, cell signaling networks, the micro-environment, and epigenetic modifications. Thus, we can link the si-VDAC1-induced reprograming of the tumor to changes in the interactions between metabolism and the regulation of cell-specific transcriptional networks mediated by an epigenetics program. Such a program involves chromatin-modifying enzymes, whose activity is directly dependent on metabolites such as acetyl-CoA, SAM, and NAD+, among others. Moreover, our results clearly demonstrate the interplay between metabolism and epigenetics, with VDAC1 silencing altering the expression of epigenetic-related factors, as revealed by DNA microarray, proteomics, immunoblotting, and qRT-PCR. DNA microarray data identified about 95 epigenetic-related genes whose expression was altered, including genes encoding histone acetyltransferases, histone deacetylases, histone and other protein methyltransferases, and demethylases (Table 1 and Table 2). Proteomics analysis revealed changes in the levels of additional proteins associated with histone acetylation and deacetylation, as well as factors controlling histone transcription (Table 3).

### 4.2. VDAC1 Depletion Altered Histones Acetylation and Methylation

Histone acetylation is an epigenetic modification that is unequivocally associated with an increase in the propensity for gene transcription. The modification is regulated by the action of histone acetyltransferases (HATs) and histone deacetylases (HDAC). In general, we found that acetylation of H3 in several sites was highly increased (Figure 4), as well as alterations in the expression of acetyltransferase, with up-regulation of KAT2A, KAT5, and KAT7 and down-regulation of KAT2B (Table 2). KAT2B is associated with cell proliferation and transcriptional activation promotion [20].

Before further discussing the obtained results, it should be mentioned that the relationship between mRNA and protein abundances is influenced by many factors. These include post-transcriptional mechanisms and the dynamic processes involved in protein synthesis and degradation, reflected in the rate of protein synthesis, or mRNA, or protein turnover, and it is suggested that transcript levels by themselves are not sufficient to predict protein levels in many scenarios [90,91].

Our results indicate that the deacetylase HDAC2, which is highly expressed in cancer and associated with tumor de-differentiation and invasion [5], was dramatically reduced (Table 1, Figure 3B–E). HDAC2 expression was highly decreased, in agreement with it being highly expressed in cancer, and its functions being associated with tumor de-differentiation and invasion. Other deacetylases, however, such as HDAC5, HDAC7, HDAC10, Sirt1, and Sirt6 were up-regulated in si-hVDAC1-TTs (Table 1, Figure 3B–E). In contrast, the expression of acetyltransferase, and of histone acetylation diminishes the electrostatic affinity between histone proteins and DNA, thereby promoting a chromatin structure that is more permissive to gene transcription [92]. For example, acetylation of K9 and K27 on histone H3 (H3K9ac and H3K27ac) is normally associated with enhancers and promoters of active genes. Thus, the changes in chromatin remodelers observed after depletion of VDAC1 is predicted to have a dramatic effect on cellular transcription, as reflected by the altered expression of over 5000 genes [49] and tumor reprograming and growth inhibition.

With respect to apparent contradictory results of HDAC2 expression being decreased, while other deacetylases such as HDAC5, HDAC7, and HDAC10 were up-regulated. HDACs were shown to form multi-protein complexes, with the acetyltransferase activity of the complex being dependent on the partners [93]. It is possible that the complexes’ composition is altered upon VDAC1 depletion, and accordingly their acetyltransferase. In addition, HDAC1 and HDAC2 activities were shown to be regulated by post-translation modifications (PTMs) [94]. Thus, in addition to these types of regulation, the epigenetic processes are also regulated by the substrates and enzyme modulators, pointing to the complicity of the epigenetic landscape. Demonstration of these changes upon VDAC1 depletion requires further investigation using complicated studies. 

Conserved histone lysine methylation, methyltransferases, demethylases, methyl-lysine-binding proteins and, thus, mis-regulation of histone lysine methylation have been implicated in reprogramming and cancer development of several cancers and developmental defects [95]. Our results show that methylated histones such as of H4, H3K9me2, H3K4m3, H3K27me2, H3K27m3, and H4K20m3 were highly reduced (Figure 3 and Figure 4) after VDAC1 depletion. The methylated sites on the histones found within heterochromatin (H3K9, H3K27, H3K79, and H4K20) demarcate subdomains; tri-methylated H3K9 and tri-methylated H4K20 are enriched in pericentric heterochromatin, whereas tri-methylated H3K27 is enriched at the inactive X-chromosome [95,96]. In addition, several methylation-related genes were up- or down-regulated in si-hVDAC1-TTs, relative to si-NT (Figure 3).

Most methylation marks characterized to date have been shown to have a role in transcription with some being activators or repressors, as well as performing other functions. This can explain the results showing both up- or down-regulated methylation-related genes upon VDAC1 depletion.

### 4.3. SIRT1, SIRT6, and Metabolism Regulation

The sirtuins (SIRTs), are a family of highly-conserved histone deacetylases (HDACs) that are differentially expressed in several human cancers, where they display both oncogenic and tumor-suppressive properties depending on cellular context and experimental conditions [97]. SIRTs have also been shown to regulate a wide variety of cellular processes beyond transcriptional repression in varied subcellular compartments and in different cell types [97]. SIRT6 is unique in its constitutive localization to chromatin [98] and has been shown to regulate many important pathways via epigenetic mechanisms, mainly histone deacetylation. The activity of the protein itself is regulated by several different mechanisms, including p53-, AP-1- (activator protein 1), and SIRT1-mediated transcriptional control [99,100,101]. Although primarily a nuclear protein, the ability of SIRT1 to deacetylate peroxisome proliferator-activated receptor gamma coactivator-1α (PGC-1α) has been extensively implicated in metabolic control, and mitochondrial biogenesis and energy metabolism [102].

SIRT6 deacetylates H3 lysine 9 and K56 (H3K9ac and H3K56ac) [103], resulting in modulation of gene expression, telomere maintenance, and genomic stability [98]. In addition, SIRT6 has been reported to be dynamic in its chromatin binding in response to stimuli such as TNFα, thereby altering the transcriptional landscape of aging and stress-related genes [104].

The observation of up-regulation of expression of both SIRT-1 and SIRT-6 si-hVDAC1-TTs (Figure 3B–E, Table 1) is in agreement with the link between SIRT1 activity and metabolic homeostasis through the ability to deacetylate target proteins [105]. SIRT6 is also heavily implicated in metabolic regulation, and SIRT6^−^/^−^ mice die at 2–4 weeks of age due to severe accelerated aging and hypoglycemia as a result of altered rates of glycolysis, glucose uptake, and mitochondrial respiration [106,107]. In addition, SIRT6 controls the acetylation state of PGC-1A in a GCN5-dependent manner that regulates blood glucose levels [108]. SIRT6 uniquely induced an increase in the acetylation of PGC-1 α through the direct modification and activation of GCN5 (also known as KAT2A), where KAT2A coupled with the α-KGDH complex acts as a histone H3 succinyltransferase. Thus, the dramatic increase in SIRT1 and SIRT6 expression in si-hVDAC1-TTs reflects the function of sirtuins as NAD^+^-dependent deacetylases in the metabolism–epigenetics link, [109].

Due to the correlations found between several human diseases and the histone acetylation balance, proteins that mediate histone acetylation have become attractive drug targets. However, HDAC inhibitors show poor selectivity for class I, II, and IV HDACs [110]. While sirtuin activators and inhibitors have also been reported, they too display only modest selectivity [111].

### 4.4. VDAC1 Depletion Altered Histones Modifications, Increasing Acetylation, and Decreasing Methylation 

Histone lysine methylation is a post-translational modification affecting transcription that primarily affects histone H3 at lysines 4, 9, 14, 18, 23, 27, 36, and 79, and histone H4 at lysine 20. Unlike acetylation, lysine methylation does not alter the charge of the residue and is therefore thought to primarily modulate chromatin structure through the recruitment of distinct reader proteins that possess the ability to facilitate transcriptional activation or repression [112]. 

Histone methylation at lysine residues occurs through the introduction of mono-, di- or tri-methyl groups, which provide functional diversity to each site of methylation. For example, both mono- or tri-methylation of K4 (H3K4me1 or H3K4me3) are active marks, but H3K4me1 is found at transcriptional enhancers, while H3K4me3 is found at gene promoters. Tri-methylation of K36 (H3K36me3) is associated with transcribed regions in gene bodies. Histone modification specific to promoters is associated with H3K4me3, distal regulatory elements with H3K4me1, and the active forms of both promoters and enhancers with H3K27ac [113,114]. 

In our previous studies [49,115,116], we demonstrated that silencing VDAC1 resulted in reprogramed metabolism and led to cell differentiation. Several modulations were shown to attribute to the differentiation of cancer stem cells (CSCs) to a non-malignant phenotype [117]. These included gene activation marks of increased H3K4 methylation and loss of H3K27 acetylation, or gain of repression marks, such as H3K9me2, H3K9me3, and H3K27me3. These genes are often found in embryonic carcinoma cells and are frequently silenced by DNA hyper-methylation in adult human cancer cells [118]. In addition, the repressive chromatin marks H3K27me3 and H4K20me3 were shown to play important roles in normal and disease conditions in the context of neural stem progenitor cells where H3K27me3 is found primarily at promoters in gene-rich regions, and is closely associated with developmental regulators in embryonic stem cells, including Hox and Sox genes. Moreover, their levels were altered in the human GBM [119]. In this context, our results indicated that the modified histones H3K4me, 3H3K9me, H3K27me, H3K27me3, and H4K20me3 were strongly reduced in the si-hVDAC1-TTs (Figure 4). 

Both H3K27me3 and H4K20me3 are associated with chromatin compaction and transcriptional repression, whose dysregulation has been implicated in tumorigenesis [31]. It has been reported that the singly methylated form of H4K20 is enriched in mitotic cells, whereas the levels of H4K20me2 and H4K20me3 are increased in quiescent cells [120]. In addition, epigenetic regulation of the cell cycle by H3K27me3 and H4K20me3 during adult neurogenesis and heterogeneity in a subtype of GBM has been reported [119]. Thus, the decrease in H3K27me3 and H4K20me3 expression in si-hVDAC1-TTs is in agreement with their predicted functions. 

In addition to changes in the expression levels of histone-modifying enzymes and SIRT1 and SIRT6, TFs also play crucial roles in regulating transcription. The expression of several TFs was altered in si-hVDAC1-TTs (Table 4) [49]. These included STAT1 and STAT6, both of which function in signal transduction and activation of transcription, as well as CSRP1, which regulates processes important for development and cellular differentiation [121]. In addition, we demonstrated that si-VDAC1 tumor treatment enhanced p53 expression and reduced c-Myc and HIF-1α expression [49]. The activity of p53 is regulated by an equilibrium between acetylation and deacetylation, which is maintained by HDAC1 and SIRT1 [122]. 

## 5. Conclusions 

We demonstrated here that VDAC1 depletion leads to metabolism reprograming, that in turn regulates epigenetically-related enzymes, and hence gene transcription, leading to tumor reprograming, including growth inhibition and cell differentiation. This reprograming has an impact on gene expression, at both epigenetic landscape and transcription factor regulation levels.

## Figures and Tables

**Table 1:** si-VDAC1 alters the expression of epigenetics-processes-related enzymes associated with acetylation. Results are from DNA-microarray analysis and represent the fold change in expression in si-hVDAC1-TTs, relative to levels in si-NT-TTs. Selected genes associated with epigenetics are presented. Genes modified ≥ 2-fold with a false discovery rate < 0.05 are listed. For each gene, the gene symbol and name, linear fold change in expression, and *p*-value are indicated. Negative numbers represent down-regulation.

Enzyme	Fold Change (*p-*Value)	Function
KAT2A (Lysine acetyltransferase 2A)	2.83 (0.04)	Histone acetyltransferase (HAT) functions in the regulation of gene expression, and promotes transcriptional activation [54]. Can act both as an acetyltransferase and a succinyltransferase [30].
KAT2B (Lysine acetyltransferase 2B)	−3.63 (0.04)	HAT has significant histone acetyltransferase activity and is associated with cell proliferation and transcriptional activation promotion [55].
KAT5 (Lysine acetyltransferase 5)	2.84 (0.03)	Catalytic subunit of histone acetyltransferase complex which is involved in transcriptional activation of select genes, principally by acetylation of nucleosomal histones H4 and H2A [56]. Regulates cell growth and proliferation [57].
KAT7 (Lysine acetyltransferase 7)	2.15 (0.04)	Part of the acetyltransferase complex. Involved in transcription and DNA replication via acetylation of histones H3 and H4 [58]
HDAC2 (Histone deacetylase 2)	−3.063 **(0.04)**	Highly expressed in cancer, associated with tumor de-differentiation and invasion [59].
HDAC5 (Histone deacetylase 5)	**4.747** **(0.05)**	Possesses deacetylase activity for histone lysine residues and other proteins (p53?) [60]. Associated with cell differentiation and is a negative regulator of cell migration and angiogenesis [61]. Represses transcription when tethered to a promoter. It also interacts with myocyte enhancer factor-2 (MEF2) protein, resulting in repression of MEF2-dependent genes [62].
HDAC7 (Histone deacetylase 7)	**3.793** **(0.03)**	Has little intrinsic deacetylase activity but may serve various other functions related to suppression of development, proliferation, and inflammation [63]. Promotes repression mediated via the transcriptional co-repressors SMRT and MEF2(A) [64].
HDAC10 (Histone deacetylase 10)	**4.323** **(0.04)**	Acts as a suppressor of cancer metastasis and cell cycle regulation [65]. Low expression of HDAC10 associated with poor prognosis of cancer [66].
SIRT1 (NAD^+^-dependent protein deacetylase sirtuin-1)	**3.307** **(0.03)**	NAD^+^-dependent protein linked to cellular survival pathways by virtue of maintaining the tumor suppressor gene p53 [67].
SIRT6 (NAD^+^-dependent protein deacetylase sirtuin-6)	**4.433** **(0.03)**	NAD^+^-dependent protein deacetylase of histone H3K9 and H3K56. Plays various roles in metabolism, stress resistance and lifespan [68,69].

**Table 2:** si-VDAC1 alters the expression of epigenetics-processes-related enzymes associated with methylation. Results are from DNA-microarray analysis and represent the fold change in expression in si-hVDAC1-TTs, relative to the levels in si-NT-TTs. Selected genes associated with epigenetics are presented. Genes modified ≥ 2-fold with a false discovery rate < 0.05 are listed. For each gene, the gene symbol and name, linear fold change in expression, and *p*-value are as indicated. Negative numbers represent down-regulation.

Enzyme	Fold Change (*p-*Value)	Function
KMT2B (Lysine methyltransferase 2B)	**3.6** (0.03)	Histone methyltransferase. Methylates histone H3K4, involved in transcriptional activation [70].
SETD6 (SET-domain-containing 6)	**3.06** (0.03)	Methyltransferase that adds a methyl group to histone H2A, which is involved in nuclear receptor-dependent transcription [64], leading to down-regulation of NF-κB transcription factor activity [71].
SETD7 (SET-domain-containing Lysine methyltransferase 7)	**−3.4** (0.03)	Histone methyltransferase that specifically mono-methylates H3K4. Implicated in multiple signaling and disease-related pathways, with a broad diversity of reported substrates [72].
SUZ12 (Polycomb repressive complex 2 subunit)	**−5.7** (0.02)	Component of the polycomb complex. Methylates H3K27, leading to transcriptional repression of the affected target gene [73,74].
JMJD1C (Jumonji-domain-containing 1C)	**−5.7** (0.03)	A candidate histone demethylase (H3K9) thought to be a co-activator of key transcription factors [75].
DNMT3A (DNA methyltransferase 3A)	**4.854** **(0.03)**	Mediates genome-wide de novo methylation and establishment of DNA methylation patterns during development. Recruited to tri-methylated H3K36 [76]. Critically important new tumor suppressor [77].
DNMT1 (DNA methyltransferase 1)	**−3.918** (0.08)	Methylates CpG residues. Mediates transcriptional repression by direct binding to HDAC2 in association with DNMT3B and dimethylation of promoter histone H3 at H3K4 and H3K9 [78].
KDM4B (Lysine demethylase 4B)	**3.685** **(0.03)**	Histone demethylase that specifically demethylates H3K9. Contributes to the regulation of cellular differentiation and proliferation [79].
KDM1A (Lysine demethylase 1A)	**−2.559** (0.04)	Histone demethylase demethylates both H3K4me and H3K9me/me2 of histone H3. Thereby, acting as a coactivator or a corepressor, depending on the context [80].
KDM6A (Lysine demethylase 6A)	**3.127** **(0.03)**	Histone demethylase that specifically demethylates H3K27me2/me3. Plays a role in cell differentiation [81].
KDM6B (Lysine demethylase 6B)	**5.775** **(0.03)**	Histone demethylase that specifically demethylates H3K27me2/me3. Plays a role in cell differentiation [81].
MTRR (Methionine synthase reductase)	**−5.629** (0.03)	Involved in the reductive regeneration of co-factor vitamin B12 required for the maintenance of methionine synthase in a functional state. Necessary for utilization of methyl groups for DNA methylation [81].

**Table 3:** Histones and histone-associated proteins differentially expressed in si-NT-TTs and si-hVDAC1-TTs, as identified by LC-HR MS/MS. LC-HR MS/MS experiments were performed as described in the Materials and Methods section. Proteins differentially expressed between si-NT-TTs and si-hVDAC1-TTs (*p*-value < 0.01, fold change ≥|2|) are presented, along with the name, fold change and *p*-value, as well as function.

Proteomics: Histones and Histone-Associated Proteins		
Protein	Fold Change (*p*-value)	Function
HPF1 (Histone PARylation factor 1)	**−14.886** (0.03)	Promotes histone serine ADP-ribosylation in response to DNA damage, limiting DNA damage-induced PARP1 hyper-auto-modification, thus ensuring genome stability.
BZW1 (Basic leucine zipper and W2 domain-containing protein 1)	**−2.109** **(0.02)**	Enhances histone H4 gene transcription.
CBX5 (Chromobox protein homolog 5)	**−9.244** (0.04)	Component of heterochromatin that recognizes and binds histone H3 tails methylated at K9 (H3K9me), leading to epigenetic repression.
HAT1 (Histone acetyltransferase type B catalytic subunit)	**−3.833** (0.01)	Acetylates soluble but not nucleosomal histone H4 at K5 and K12 and, to a lesser extent, acetylates H2AK5.
HDAC3 (Histone deacetylase 3)	**−2.284** (<0.01)	Responsible for the deacetylation of lysine residues in the N-terminal region of the core histones. Participates in BCL6 transcriptional repressor activity by deacetylating H3K27 on enhancer elements.
H2AFZ (H2A histone family member Z)	**−2.7** (0.02)	Variant histone H2A which replaces conventional H2A in a subset of nucleosomes.
HP1BP3 (Heterochromatin protein 1-binding protein 3)	**−2.103** (0.03)	Component of heterochromatin that maintains heterochromatin integrity during G1/S progression and regulates the duration of G1 phase to critically influence cell proliferative capacity. Mediates chromatin condensation during hypoxia, leading to increased tumor cell viability, radio-resistance, chemo-resistance, and self-renewal.
H1F0 (Histone H1.0)	**2.548** (<0.01)	Histones H1 are necessary for the condensation of nucleosome chains into higher-order structures. H1F0 histones are found in cells in terminal stages of differentiation or those that have low rates of cell division.

**Table 4:** Transcription factors differentially expressed in si-NT-TTs and si-hVDAC1-TTs, as identified by LC-HR MS/MS. LC-HR MS/MS experiments were performed as described in the Materials and -Methods section. Proteins differentially expressed between si-NT-TTs and si-hVDAC1-TTs (*p*-value < 0.01, FC ≥ |2|) are presented, along with the name, fold change, and *p*-value, as well as function.

Proteomics: Transcription Factors		
Protein	Fold Change (*p*-Value)	Function
LARP7 (La-related protein 7)	**−127.546** (0.04)	Negative transcriptional regulator of polymerase II genes.
MATR3 (Matrin-3)	**−4.372** (<0.01)	May play a role in transcription or interact with other nuclear matrix proteins to form the internal fibrogranular network.
TOP1 (DNA topoisomerase 1 alpha)	**−4.154** (0.04)	Releases the supercoiling and torsional tension of DNA introduced during replication and transcription by transiently cleaving and rejoining one strand of the DNA duplex.
SMARCD2 (SWI/SNF-related matrix-associated actin-dependent regulator of chromatin subfamily D member 2)	**−3.63** (0.04)	Involved in transcriptional activation and repression of select genes by chromatin remodeling (alteration of DNA–nucleosome topology).
SUB1 (Activated RNA polymerase II transcriptional coactivator p15)	**−3.481** (0.03)	General co-activator that cooperatively functions with tumor associated fibroblasts (TAFs) and mediates functional interactions between upstream activators and the general transcriptional machinery.
RALY (RNA-binding protein Raly)	**−2.3** (0.02)	RNA-binding protein that acts as a transcriptional co-factor for cholesterol biosynthetic genes in the liver.
CNOT9 (CCR4-NOT transcription complex subunit 9)	**−1.9** (<0.01)	Component of the CCR4-NOT complex, which is one of the major cellular mRNA deadenylases and is linked to cellular processes, including bulk mRNA degradation, miRNA-mediated repression, translational repression during translational initiation, and general transcription regulation.
STAT1 (Signal transducer and activator of transcription 1-alpha/beta)	−1.7 (0.03)	Signal transducer and transcription activator that mediates cellular responses to interferons (IFNs), other cytokines, and other growth factors.
CSRP1 (Cysteine- and glycine-rich protein 1)	**4.8766** (<0.01)	May be involved in regulatory processes important for development and cellular differentiation.
ETS1 (ETS proto-oncogene 1)	5 (0.02)	Transcription factor. Directly controls the expression of cytokine and chemokine genes in a wide variety of different cellular contexts. May control the differentiation, survival, and proliferation of lymphoid cells. May also regulate angiogenesis by regulating the expression of genes controlling endothelial cell migration and invasion.
STAT6 (Signal transducer and activator of transcription 6)	11.5 (0.01)	Carries out dual functions, affecting signal transduction and the activation of transcription.

## Supplementary Materials

The following are available online at https://www.mdpi.com/2072-6694/12/4/1031/s1: Methods, Figure S1: Volcano plot of genes with altered expression, Figures S2 and S3: Specificity of the antibodies used to follow histones and their acetylated and methylated versions, Table S1: Antibodies used in this study, and Table S2: primers for Q-RT- PCR used in this study.
