# Supplementary material for: The Mitochondrial Protein VDAC1 at the Crossroads of Cancer Cell Metabolism: The Epigenetic Link
Source: Cancers (Basel). 2020 Apr 22;12(4):1031. doi: 10.3390/cancers12041031 (PMC7226296; doi:10.3390/cancers12041031)
Supplement: Supplementary file 1 [file cancers-12-01031-s001.pdf]

Supplementary Materials: The Mitochondrial Protein VDAC1 at the Crossroads of Cancer Cell Metabolism: The Epigenetic Link

Zohar Amsalem, Tasleem Arif, Anna Shteinfer-Kuzmine, Vered Chalifa-Caspi and Varda Shoshan-Barmatz

Methods

Immunohistochemistry (IHC)

Immunohistochemical staining was performed on formalin-fixed and paraffin-embedded tumors obtained from si-NT- and si-hVDAC1-treated tumors as described previously [1]. Sections were deparaffinized using xylene and a graded ethanol series. Endogenous peroxidase activity was blocked by incubating the sections in 3% H_2O_2 for 10 minutes. Antigen retrieval was performed in 0.01M citrate buffer (pH 6.0) at 95–98 °C for 20 min. After washing sections in PBS (pH 7.4), non-specific antibody binding was reduced by incubating the sections in 10% normal goat serum for 2 h. After decanting excess serum, sections were incubated overnight at 4 °C with primary antibodies (Table S1). After washing with PBS, the sections were incubated for 2 h with the appropriate secondary antibodies conjugated to horseradish peroxidase (Table S1). Sections were washed three times in PBS and subsequently, the peroxidase-catalyzed reaction was visualized by incubating with 0.02% DAB. After rinsing in water, the sections were counterstained with hematoxylin, and mounted with Vectashield mounting medium (Vector Laboratories, Burlingame, CA, USA). Finally, the sections were observed under a microscope (DM2500, Leica, Wetzlar, Germany) and images were captured at the indicated magnification with the same light intensity and exposure time. Controls were carried out with the same protocols but omitting the primary antibodies.

RNA Preparation and DNA Microarray Analysis

Total RNA was isolated from si-Scr- and si-hVDAC1-treated tumors (4-6 mice each) using the RNeasy mini kit (Qiagen) according to the manufacturer's instructions. Total RNA quality was analyzed using the Agilent RNA 6000 nano kit. The RNA integrity values obtained for total RNA extracted from si-Scr- and si-hVDAC1-treated tumors were 8 to 10. The targets for Affymetrix whole transcript expression microarray analyses were prepared using the Affymetrix GeneChip WT PLUS reagent kit according to the manufacturer's instructions and hybridized to Human Gene 1.0 ST microarrays. Data were acquired using the Affymetrix GeneChip algorithm (version 3.2). Files were imported to Partek Genomics Suite, and all probes except control probes were pre-processed by RMA background correction, log2 transformation and probe set summarization using median polish. Probe sets with signals < 5 in all samples were filtered out. Subsequently, global scaling was carried out by shifting the mean of each sample to the grand mean (i.e. in each sample, the mean signal was subtracted from each of the signals, and then the grand mean of all the samples was added, such that all arrays eventually had the same mean signal). Differentially expressed genes were defined as those having FDR-adjusted t test p-value < 0.05, and two clusters were defined, up-regulated and down-regulated genes (linear fold change > 2 or < -2, respectively). Each cluster was tested separately for enrichment of functional groups based on the GO system.

Quantitative Real-Time PCR (q-RT-PCR)

Real-time RT-PCR was performed (KiCqStart Primers; Sigma Aldrich, St. Louis, Missouri, USA) in triplicate, using the Power SYBR green master mix (Applied Biosystems, Foster City, CA, USA). Genes examined and primers used are listed in Table S2.Levels of target genes were normalized relative to β-actin mRNA levels. Samples were amplified by a 7300 Real Time PCR System (Applied Biosystems) for 40 cycles using the following PCR parameters: 95 °C for 15 seconds, 60 °C for 1 minute,

and 72 °C for 1 minute. The copy numbers for each sample were calculated by the CT-based calibrated standard curve method. The mean fold changes (± SEM) of the three replicates were calculated.

Gel Electrophoresis and Immunoblotting

To extract proteins for immunoblotting, tumor tissues were solubilized in a lysis buffer (50 mM Tris-HCl, pH 7.5, 150 mM NaCl, 1 mM EDTA, 1.5 mM $MgCl_2$, 10% glycerol, 1% Triton X-100, a protease inhibitor cocktail (Calbiochem)), followed by sonication and centrifugation (10 min, 600 g). The protein concentration of each lysate was determined using a Lowry assay. Samples were stored in −80 °C until analysis by gel electrophoresis and immunoblotting, as described in Supplementary Data.

For immunostaining, membranes containing electro-transferred proteins following SDS-PAGE were blocked with 5% non-fat dry milk and 0.1% Tween-20 in TBS, incubated with the primary antibodies (sources and dilutions as detailed in Table S1) and then with HRP-conjugated anti-mouse or anti-rabbit (1:10,000) or anti-goat (1:20,000) IgG. Enhanced chemiluminescent substrate (Pierce Chemical, Rockford, IL, USA) was used to detect HRP activity. Band intensities were analyzed by densitometry using FUSION-FX (Vilber Lourmat, France) software, and values were normalized to the intensities of the appropriate β-actin signal that served as a loading control.

LC-HR MS/MS Analysis

Tumors from three mice treated with si-NT or si-hVDAC1 were subjected to in-solution tryptic digestion as follows. Proteins were first reduced by incubation with 5 mM DTT for 30 min at 60 °C, followed by alkylation with 10 mM iodoacetamide in the dark for 30 min at 21 °C. Proteins were then subjected to digestion with trypsin (Promega, Madison, WI, USA) at a 1:50 trypsin:protein ratio for 16 h at 37 °C. Following digestion, detergents were cleared from the samples using commercial detergent removal columns (Pierce, Rockford, IL, USA), and desalted using solid-phase extraction columns (Oasis HLB, Waters, Milford, MA, USA). Digestions were stopped by addition of trifluroacetic acid (1%). The samples were stored at −80 °C until LC-HR MS/MS analysis.

For LC-HR MS/MS, ULC/MS grade solvents were used for all chromatographic steps. Each sample was separated using split-less nano-ultra performance liquid chromatography columns (10K psi nanoAcquity; Waters). The mobile phase was (A) H_2O and 0.1% formic acid, and (B) acetonitrile and 0.1% formic acid. Desalting of the samples was performed online using a reverse-phase C18 trapping column (180 μm internal diameter, 20 mm length, 5 μm particle size; Waters). The peptides were then separated on a T3 HSS nano-column (75 μm internal diameter, 250 mm length, 1.8 μm particle size; Waters) at 0.3 μL/min. Peptides were eluted from the column into the mass spectrometer using the following gradient: 4% to 35% (B) for 150 min, 35% to 90% (B) for 5 min, maintained at 90% for 5 min and then back to initial conditions. The nano-UPLC was coupled online through a nano-ESI emitter (10 μm tip; New Objective, Woburn, MA, USA) to a quadrupole Orbitrap mass spectrometer (Q Executive, Thermo Scientific, Waltham, MA, USA) using a FlexIon nanospray apparatus (Proxeon, Odense, Denmark). Data was acquired in the DDA mode, using a Top12 method [2]. Raw data was imported into Expressionist software (Genedata, Switzerland) [3,4]. The software was used for retention time alignment and peak detection of precursor peptide intensities. A master peak list was generated from all MS/MS events and sent for database searching using Mascot v2.4 (Matrix Sciences, Maharashtra, India). Data was searched against a database containing forward and reverse human protein sequences from UniprotKB/SwissProt, and 125 common laboratory contaminants, totaling 20,304 entries. Fixed modification was set to carbamidomethylation of cysteines, while variable modification was set to oxidation of methionines. Search results were then imported back to Expressionist for annotation of detected peaks. Identifications were filtered such that the global false discovery rate was a maximum of 1%. Protein abundance was calculated based on the three most abundant peptides [5]. Proteins with less than 2 unique peptides were excluded from further analysis.

LC-HR-MS/MS data were imported into Partek Genomics Suite software (Partek, St. Louis, MO, USA) and differences between expression levels of the proteins in the different groups were

calculated using a *t*-test. Functional enrichment analysis of differentially expressed proteins was performed using the DAVID and Gene Ontology (GO) bioinformatics resources, v6.7 [6].

Results

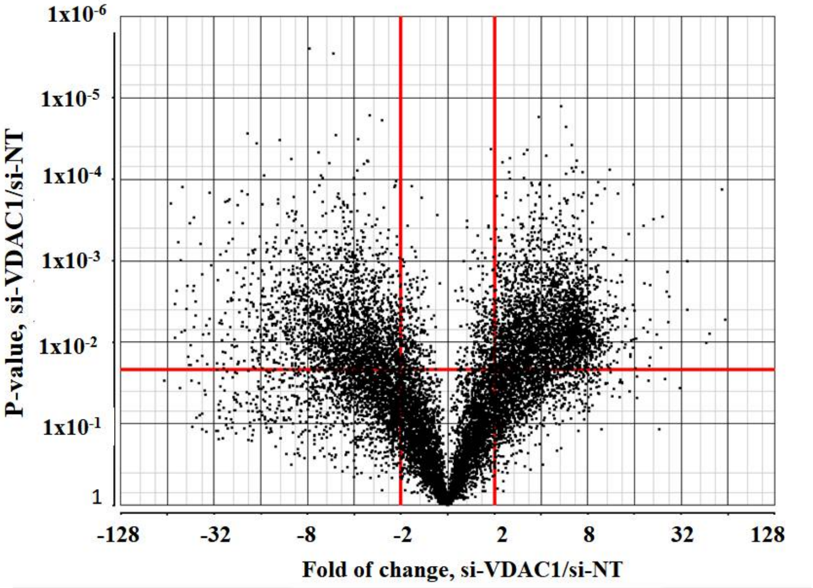

Figure S1. Volcano plot of genes with altered expression. Volcano plot of all genes showing p-value as function of the fold change in the si-hVDAC1-TTs relative to si-NT-TTs. Cutoff lines are at FDR-adjusted *p*-value = 0.05 and linear fold change of decreased or increased expression ≥ −2 and 2, respectively in the si-hVDAC1-TTs relative to si-NT-TTs. Total number of differentially expressed genes was 5,271 of which 2,291 were down-regulated transcripts and 2,980 were up-regulated transcripts.

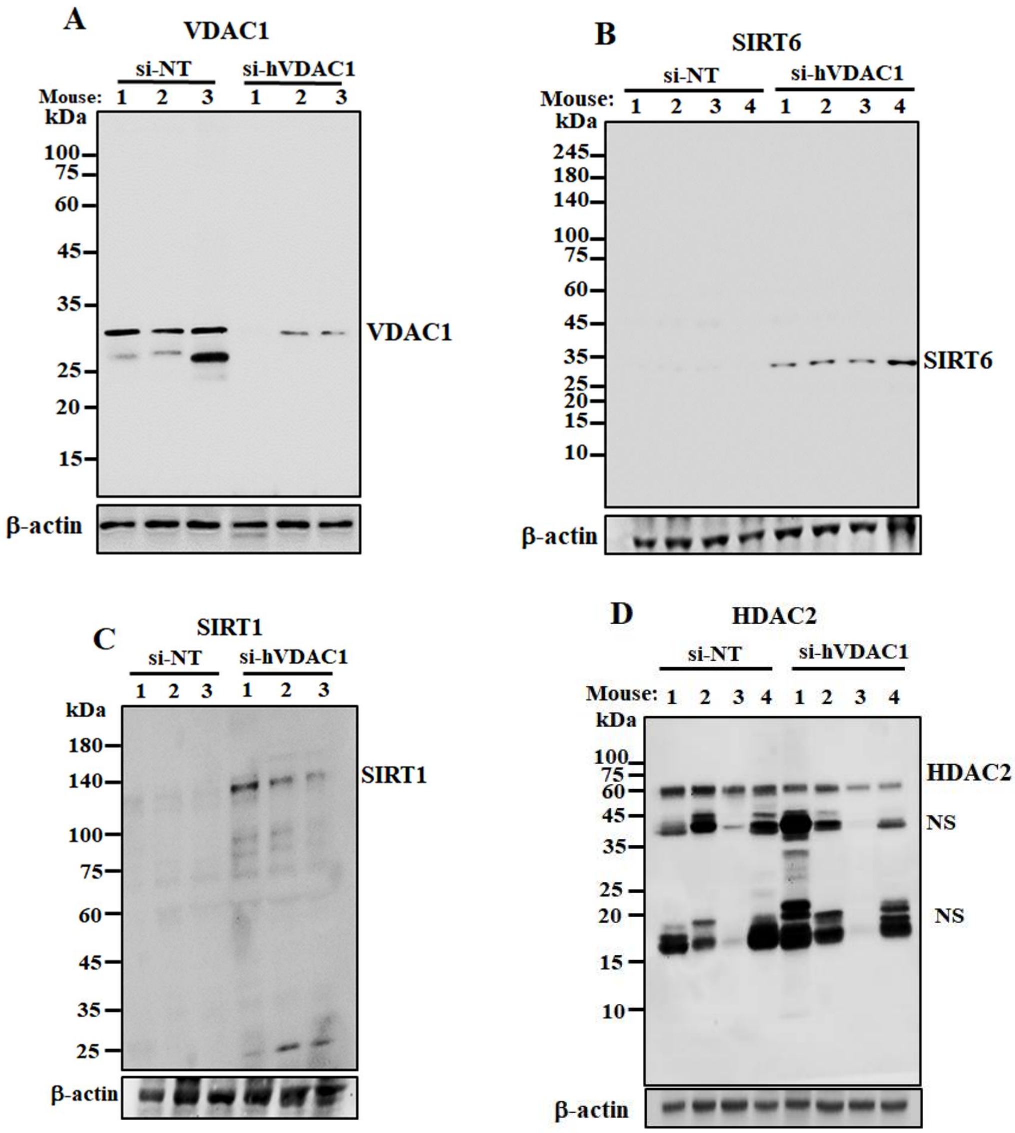

Figure S2. Whole blots for Figures 1C and 3C. Demonstrating the reactivity of the antibodies used to follow VDAC1 (**A**), SIRT6 (**B**), SIRT1 (**C**) and HDAC2 (**D**), using specific antibodies with the complete blots are shown to demonstrate antibodies specificity. Proteins were isolated from si-NT-TTs and si-hVDAC1-TTs in the presence of deacetylation inhibitors as described in the Methods. β-actin served as an internal loading control. The positions of molecular weight protein standards are provided. NS, indicates non-specific.

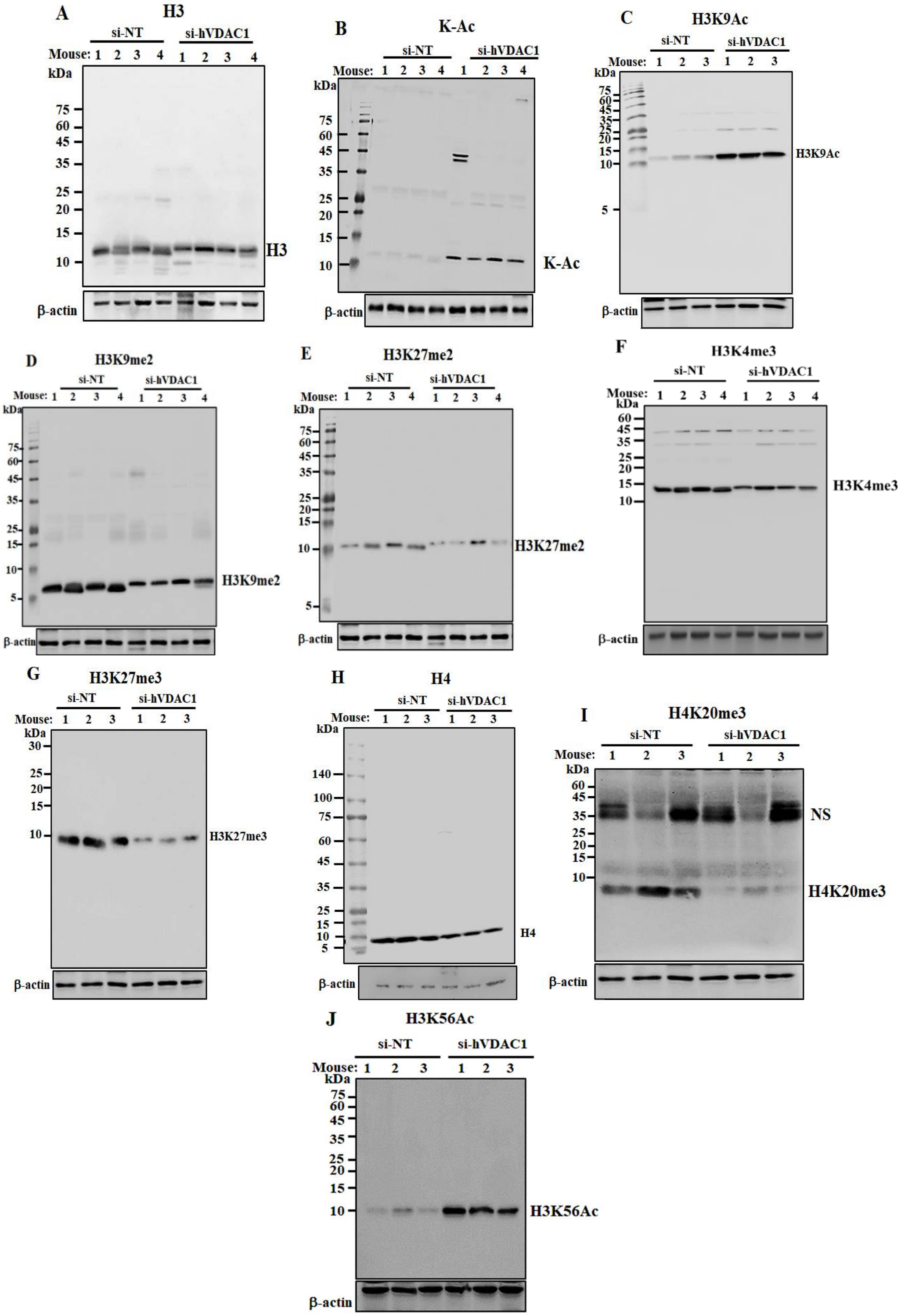

Figure S3. Whole blots for Figure 4A-D. Demonstrating the reactivity of the antibodies used to follow histones and their acetylated and methylated versions. Proteins were isolated from si-NT-TTs and si-hVDAC1-TTs in the presence of deacetylation inhibitors as described in the Methods. Immunoblots of: histone 3 (H3) (**A**), acetylated histones (K-Ac) (**B**), H3K9Ac (**C**) H3K9me2 (**D**), H3K27me2 (**E**), H3K4me3 (**F**), H3K27me3 (**G**), histone 4 (H4) (**H**), H4K20me3 (**I**), and H3K56Ac (**J**) using specific antibodies with the complete blots are shown to demonstrate antibodies specificity. β actin served as an internal loading control. The positions of molecular weight protein standards are provided. NS indicates non-specific.

Table S1. Antibodies used in this study. Antibodies against the indicated protein, their catalogue number, source, and the dilutions used in immunoblot and immunofluorescence experiments are presented.

Antibody	Source and Cat. No.	Dilution	
		IHC	WB
Rabbit monoclonal anti-VDAC1	Abcam, Cambridge, UK, ab154856	-	1:5000
Mouse monoclonal anti-actin	Millipore, Billerica, MA, MAB1501	-	1:40000
Rabbit monoclonal anti-Glut1	Abcam, Cambridge, UK ab40084	1: 200	-
Mouse monoclonal anti-GAPDH	Abcam, Cambridge, UK, ab9484	1: 200	-
Rabbit polyclonal anti-citrate synthase	Abcam, Cambridge, UK ab96600	1:200	-
Rabbit monoclonal cytochrome c oxidase subunit IVc	Abcam, Cambridge, UK, ab150422	1:200	-
Mouse monoclonal anti-ATP5a	Abcam, Cambridge, UK, ab14748	1:300	-
Rabbit polyclonal anti-H3	Abcam, Cambridge, UK, ab18521	-	1:20000
Rabbit polyclonal anti-H4	Abcam, Cambridge, UK, ab10158		1:3000
Rabbit anti-acetyl lysine	ImmuneChem Pharmaceuticals Inc.,burnaby,Canada, ICP0380	-	1:1000
Rabbit monoclonal anti-Di-methyl-histone H3K27	Cell Signaling Technology, Danvers, USA, mAb#9728	-	1:1000
Mouse monoclonal anti-H3k27me3	Abcam, Cambridge, UK, ab6002	-	1:10000
Mouse monoclonal anti- di-methyl H3 (lysine 9)	Abcam, Cambridge, UK, ab1220	-	1:1000
Rabbit monoclonal anti- histone H3 (acetyl K9)	Abcam, Cambridge, UK, ab177177	-	1:5000
Anti-Histone H3 (acetyl K56)	Abcam, Cambridge, UK, ab76307	-	1:1000
Rabbit polyclonal anti-H3K4me3	Abcam, Cambridge, UK, ab8580	-	1:2000
Rabbit polyclonal anti-H4K20me3	Abcam, Cambridge, UK, ab9053	-	1:2000
Rabbit polyclonal anti-Sirt1	Millipore, Billerica, MA, 07-131	-	1:2500
Rabbit polyclonal anti-Sirt6	Abcam, Cambridge, UK, ab62739	-	1:5000
Mouse monoclonal anti-HDAC2	Santa Cruz Biotechnology, Inc. Dallas, TX, sc9959	-	1:5000
Goat anti-Rabbit	KPL, Gaithersburg, USA, 474-1506	-	1:15,000
Goat anti-Mouse	Abcam, Cambridge, UK, ab97040	-	1:10,000

Table S2. Primers for Q-RT-PCR used in this study. The genes examined, and the forward and reverse sequences of the primers used are indicated.

Gene	Primer Sequences
ß-Actin	Forward 5′-ACTCTTCCAGCCTTCCTTCC-3′<br>Reverse 5′-TGTTGGCGTACAGGTCTTTG-3′
GLUT1	Forward 5′-GGCCATCTTTTCTGTTGGGG-3′<br>Reverse 5′-TCAGCATTGAATTCCGCCG-3′
GAPDH	Forward 5′-TGGAAGGACTCATGACCACA-3′<br>Reverse 5′-ATGATGTTCTGGAGAGCCCC-3′
CS	Forward 5′-AGGAACAGGTATCTTGGCTCT-3′<br>Reverse 5′-GGGGTGTAGATTGGTGGGAA-3′
ATP synthase 5a.	Forward 5′-TCAGTCTACGCCGCACTTAC-3′<br>Reverse 5′-GACATCTCAGCAGTCCCACA-3′
VDAC1	Forward 5′-AATGACGGGACAGAGTTTGG<br>Reverse 5′-AGCGCGTGTTACTGTTTCCT
LDH-A	Forward 5′-GCAGGTGGTTGAGAGTGCTT-3′<br>Reverse 5′-GCACCCGCCTAAGATTCTTC-3′
HDAC5	Forward 5′-TCTTGACATCACCGCAGCTC-3′<br>Reverse 5′-GACCTGACATCCCATCCGAC-3′
HDAC7	Forward 5′-CAATGTCAATGTGGCCTGGG-3′

	Reverse 5′-TCCAGCAGACACCAGGACTA-3′
DNMT1	Forward 5′-CGTGGAAGCCGGCAAAG-3′<br>Reverse 5′-GTTGTGCTGAAGAAGCCGTC-3′
KDM6B	Forward 5′-GGACTCCAGCGTTTCACCAG-3′<br>Reverse 5′-GCGGGAGTTTGGTAATGGTC-3′
SIRT1	Forward 5′-CCCTCAAAGTAAGACCAGTAGC-3′<br>Reverse 5′-GCAGATGAGGCAAAGGTTCTC-3′
HDAC10	Forward 5′-GCACAGCCCAGAGTATGTATC-3′<br>Reverse 5′-AAGGTACTCGGGTGGAAGTA-3′
KAT2A	Forward 5′-TGAGCAGGTCAAGGGTTATG-3′<br>Reverse 5′-CGTAGGTGAGGAAGTAGAGAATG-3′

References

1. Arif, T.; Krelin, Y.; Nakdimon, I.; Benharroch, D.; Paul, A.; Dadon-Klein, D.; Shoshan-Barmatz, V. VDAC1 is a molecular target in glioblastoma, with its depletion leading to reprogrammed metabolism and reversed oncogenic properties. *Neuro. Oncol.* **2017**, *19*, 951–964, doi:10.1093/neuonc/now297.
2. Kelstrup, C.D.; Young, C.; Lavallee, R.; Nielsen, M.L.; Olsen, J.V. Optimized fast and sensitive acquisition methods for shotgun proteomics on a quadrupole orbitrap mass spectrometer. *J. Proteome Res.* **2012**, *11*, 3487–3497, doi:10.1021/pr3000249.
3. Ueda, K.; Saichi, N.; Takami, S.; Kang, D.; Toyama, A.; Daigo, Y.; Ishikawa, N.; Kohno, N.; Tamura, K.; Shuin, T.; et al. A comprehensive peptidome profiling technology for the identification of early detection biomarkers for lung adenocarcinoma. *PLoS ONE* **2011**, *6*, e18567, doi:10.1371/journal.pone.0018567.
4. Guryca, V.; Lamerz, J.; Ducret, A.; Cutler, P. Qualitative improvement and quantitative assessment of N-terminomics. *Proteomics* **2012**, *12*, 1207–1216, doi:10.1002/pmic.201100430.
5. D’Arena, G.; Laurenti, L.; Capalbo, S.; D'Arco, A.M.; De Filippi, R.; Marcacci, G.; Di Renzo, N.; Storti, S.; Califano, C.; Vigliotti, M.L.; et al. Rituximab therapy for chronic lymphocytic leukemia-associated autoimmune hemolytic anemia. *Am. J. Hematol.* **2006**, *81*, 598–602, doi:10.1002/ajh.20665.
6. Nawarak, J.; Huang-Liu, R.; Kao, S.H.; Liao, H.H.; Sinchaikul, S.; Chen, S.T.; Cheng, S.L. Proteomics analysis of A375 human malignant melanoma cells in response to arbutin treatment. *Biochim. Biophys. Acta* **2009**, *1794*, 159–167.

© 2020 by the authors. Licensee MDPI, Basel, Switzerland. This article is an open access article distributed under the terms and conditions of the Creative Commons Attribution (CC BY) license (http://creativecommons.org/licenses/by/4.0/).
